# Impact of the Number of Dissected Lymph Nodes on Survival for Gastric Cancer after Distal Subtotal Gastrectomy

**DOI:** 10.1155/2011/476014

**Published:** 2011-07-24

**Authors:** Chang-Ming Huang, Jian-Xian Lin, Chao-Hui Zheng, Ping Li, Jian-Wei Xie, Jia-Bin Wang

**Affiliations:** Department of Gastric Surgery, Fujian Medical University Union Hospital, No. 29 Xinquan Road, Fuzhou, Fujian 350001, China

## Abstract

*Objectives*. To investigate the prognostic impact of the number of dissected lymph nodes (LNs) in gastric cancer after curative distal gastrectomy. *Methods*. The survival of 634 patients who underwent curative distal gastrectomy from 1995 to 2004 was retrieved. Long-term surgical outcomes and associations between the number of dissected LNs and the 5-year survival rate were investigated. *Results*. The number of dissected LNs was one of the most important prognostic indicators. Among patients with comparable T category, the larger the number of dissected LNs was, the better the survival would be (*P* < 0.05). The linear regression showed that a significant survival improvement based on increasing retrieved LNs for stage II, III and IV (*P* < 0.05). A cut-point analysis yields the greatest variance of survival rate difference at the levels of 15 LNs (stage I), 25 LNs (stage II) and 30 LNs (stage III). *Conclusion*. The number of dissected LNs is an independent prognostic factor for gastric cancer. To improve the long-term survival of patients with gastric cancer, removing at least 15 LNs for stage I, 25 LNs for stage II, and 30 LNs for stage III patients during curative distal gastrectomy is recommended.

## 1. Introduction

Although gastric cancer is a declining incidence, its mortality remains high [1]. Complete resection is currently the only treatment that can lead to cure for gastric cancer. Recently, the surgical procedures and the extent of lymphadenectomy for gastric cancer receive more recognition with the ongoing in-depth researches [2]. Comparing with total gastrectomy, distal gastrectomy raises the lower postoperative morbidity and mortality rates and improves postoperative life quality and nutrition condition. It is regarded as one of the most important operative procedures for gastric cancer [2–4]. The goal of surgery for gastric cancer is R0 resection, but the optimal extent of lymphadenectomy remains controversial [5–7]. In the 1997 UICC TNM classification (5th edition), it was recommended that 15 or more lymph nodes should be examined for accurate staging of gastric cancer [8]. However, there are fewer studies on how many nodes should be removed and examined during performing a radical distal gastrectomy for different stages [9, 10]. Therefore, the aim of this retrospective study is to evaluate the long-term effect of the number of dissected LNs on the prognosis after curative distal gastrectomy in patients with gastric cancer. 

## 2. Materials and Methods

### 2.1. Materials

1289 patients diagnosed as primary gastric cancer were treated with curative resection (R0) at the Department of Gastric Surgery, Fujian Medical University Union Hospital, Fuzhou, China, between January, 1995 and November, 2004. All the operations were undertaken according to the Japanese Classification of Gastric Carcinoma (JCGC) [11]. Most of the early gastric cancer received D1 + *α*/*β* LN dissection, while more advanced gastric cancer received a D2 LN dissection (a few of patients with advanced gastric cancer who received a D1 + *α*/*β* LN dissection were estimated as early gastric cancer or without LN metastasis before or during the operation). There were 709 patients who underwent distal gastrectomy, and 75 patients were excluded from the study for T4b lesions (T4b lesions were not included to avoid data heterogeneity, as resections in these cases were expected to be more extensive, and the likelihood for positive margins was judged to be higher). Finally, 634 patients were enrolled in this study. The curative surgery was defined as follows: (1) complete tumor removal; (2) no macroscopic residual tumor; (3) no invasion of carcinoma cells in the margin; (4) no evidence of distant metastasis. Most of nodal materials were separately dissected from the en bloc specimen at the end of the procedure by the surgeons, and the remainder nodes were identified and retrieved by specialized pathologists from formalin-fixed surgical specimens without using any specific technique to increase nodal retrieval rate. Paraffin-embedded nodes were stained with haematoxylin and eosin and examined microscopically for metastases by specialized pathologists. Staging was done according to the 7th edition of the UICC TNM classification [12]. Patients were stratified into seven groups based on the number of total LNs removed as follows: 0~9 LNs (32 cases), 10~14 LNs (75 cases), 15~19 LNs (111 cases), 20~24 LNs (161 cases), 25~29 LNs (118 cases), 30~34 LNs (78 cases), and ≥35 LNs (59 cases). Patients with advance gastric cancer received fluorouracil-based postoperative adjuvant chemotherapy, and there was no patient who underwent preoperative chemotherapy. The followup was carried out by trained investigators through mailings, telephone calls, visiting patients, or recording the patients' consultations at the outpatient service. Most patient routine followup consisted of physical examination, laboratory tests (including AFP, CA19-9, and CEA levels), chest radiography, and abdominopelvic ultrasonography or computed tomography (CT). At the early stage (pT1), patients were followed up every 6 months during the first 2 years, and then every year beyond the third year; at more advanced stages (pT2 or greater), follow-up was every 3 months during the first year, every 6 months, or yearly beyond the second year, for a total of 5 years. Endoscopy was performed every 6 months or every year. All surviving patients were followed up for more than five years. The survival time was the time from the surgical intervention until the last contact, the date of death, or the date that the survival information was collected. The median follow up for the entire cohort was 62.0 months (range, 1~172 months). The followup rate was 93.2%, with 591 cases involved. 

### 2.2. Statistical Analysis

The statistical analysis has been performed using the Statistical Package for Social Science (SPSS) 16.0. Pearson's correlation coefficient (*r*) was used for studying the relationship between the number of metastatic lymph nodes and the number of dissected lymph nodes; actuarial survival rate was determined via the Kaplan-Meier method, with univariate comparisons between groups through the log-rank test. Cox proportional hazard model was used for multivariate analyse, with a backward elimination model for all covariates. Linear projection model was applied to analysis the impact of the number of nodes removed in increments of 10 on absolute 5-year survival. Significance of differences was assumed at *P* values less than 0.05 for all analyses. 

## 3. Results

### 3.1. Clinicopathological Characteristics of Patients

Clinicopathological features of patients are summarized in Table 1. There were 467 male and 167 female patients whose ages ranged from 22 to 87 years (56.1 ± 12.1 years). The tumor diameter was 4.3 ± 1.7 cm. Patients were divided according to the primary site of gastric cancer: 521 lower-third (L) tumors; 42 lower- and middle-third (LM) tumors; 71 middle-third (M) tumors. According to the Japanese Classification of Gastric Carcinoma [11], there were 140 differentiated and 494 undifferentiated cases. Based on the UICC TNM classification (7th edition) [12], there were 118 stage pT1, 106 stage pT2, 68 stage pT3, and 342 stage pT4a tumors. A total of 439 patients (69.2%) had LN metastasis. The incidence of LN metastasis is 18.1% pN1, 18.5% pN2, and 32.6% pN3. There were 133 (21.0%) patients with stage I, 165 (26.0%) stage II, and 336 (53.0%) stage III, according to the TNM classification [12]. There were no patients with early gastric cancer who received postoperative chemotherapy, and in all 516 patients with advanced gastric cancer, 382 cases received postoperative chemotherapy, and 134 patients did not receive postoperative chemotherapy. 

### 3.2. Long-Term Surgical Outcomes

**Figure 1:**
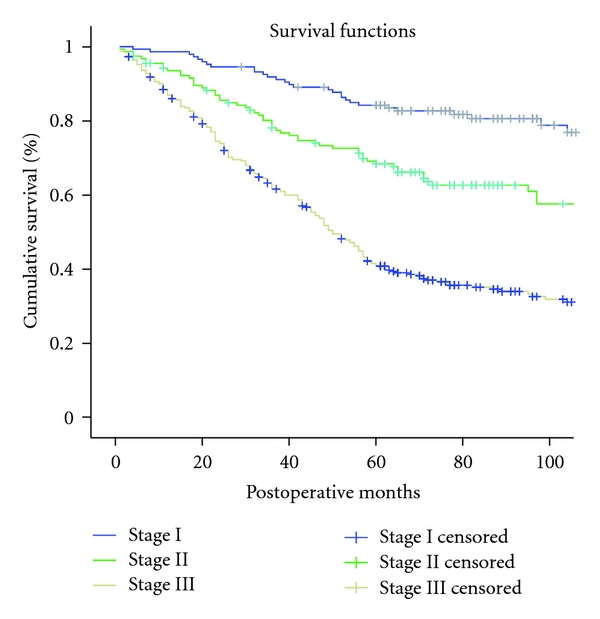
Cumulative survival curves for patients undergoing curative distal gastrectomy according to the 7th edition of the UICC TNM classification. There was significant difference between the subgroups (*P* < 0.05).

The five-year overall survival rate of the entire cohort was 57.6% in this study. Survival rate at 5 years was 85.0%, 66.0%, and 42.5% for stages I, II, and III disease, respectively (*P* < 0.05; Figure 1). 

### 3.3. Analysis Based on Lymph Node Metastasis and Retrieved Nodes

**Figure 2:**
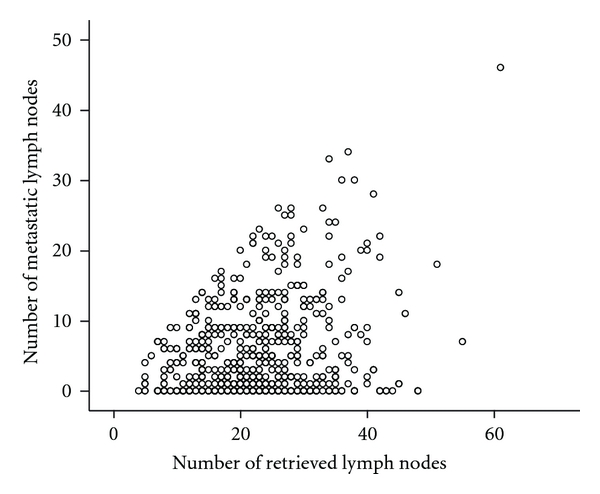
Pearson's correlation tests. Significant correlation between the number of lymph node metastases and retrieved lymph nodes (*r* = 0.252, *P* < 0.001).

Overall, 69.2% of patients (439/634) had LN metastasis. The median of total LN number was 23 (range, 5–61; mean 23.1 ± 8.6) per patient, and the median of positive LNs was 6 (range, 1–46) per patient. There was a significant correlation between the number of metastatic LNs and retrieved nodes according to the Pearson's correlation test (*r* = 0.252, *P* < 0.001; Figure 2). 

### 3.4. Univariate and Multivariate Survival Analysis

The clinicopathological variables tested in the univariate analysis were shown in Table 2. The factors influencing the 5-year survival rate were tumor diameter (*P* = 0.036), pathological types (*P* = 0.003), depth of invasion (*P* < 0.001), pN category (*P* < 0.001), and number of dissected LNs (*P* < 0.001). The covariates gender (*P* = 0.991), age (*P* = 0.423), tumor location (*P* = 0.058), adjuvant chemotherapy (*P* = 0.117), and digestive tract construction (*P* = 0.064) each had no significant influence on the survival. Multiple survival analysis was calculated by the Cox's proportional hazard regression model. The prognostic factors considered on univariate analysis were analyzed first by the stepwise regression, including tumor diameter, pathology types, depth of invasion, pN category, and the number of dissected LNs. As a result, there were three independent, statistically significant prognostic parameters: depth of invasion (*P* < 0.001), pN category (*P* < 0.001), and the number of dissected LNs (*P* < 0.001). The 95% confidence intervals were listed in Table 3. 

### 3.5. Impact of Total LN Counts on Five-Year Survival by Stage Subgroup

The overall survival results by stage subgroup and number of LNs examined were depicted in Table 4. Among patients with comparable T category, the overall survival rate was significantly different and always in favor of the higher LN count. For example, for the pT1 subgroup, 5-year survival rate increased from 62.5% (0 to 9 LNs examined) to 100% (>35 LNs examined). A similar trend was encountered for the other subgroups. 

### 3.6. Projected Numeric LN Impact on Overall Survival

Based on the statistical linearity regression, the impact of the number of dissected LNs on overall survival was calculated. The hypothetical baseline 5-year survival with an assumed zero LN examined was 43.5% for stage I, 13.6% for stage II, 2.0% for stage III, and 20.4% for the entire cohort. For every 10 extra LNs added to the total LN count, the calculated overall survival rate increased by 13.9% (stage I), 18.7% (stage II), 15.5% (stage III), and 13.1% (the entire cohort). In this setting, the regression model showed a statistically significant survival improvement based on increasing total LN counts for patients with stages II, III, and the entire cohort (*P* < 0.05; Table 5). 

### 3.7. Cut-Point Analysis of Survival

A cut-point analysis was performed to identify the numeric LN value that determines the obvious actuarial survival difference between subgroups. Univariate survival results by stage subgroup and total LN counts are depicted in Table 6. For the stage I subgroup, it was found that there were significant survival differences among the patients with 0~9 LNs, 10~14 LNs, and 15~19 LNs (*P* < 0.05). However, there were not survival differences among patients with 15~19 LNs, 20~24 LNs, 25~29 LNs, 30~34 LNs, and ≥35 LNs with univariate analysis. So we are convinced that the cutoff level was 15 LNs. In the stage II subgroup, the survival among patients with 0~9 LNs, 10~14 LNs, 15~19 LNs, 20~24 LNs, and 25~29 LNs was significantly different (*P* < 0.05). On the contrary, there were not survival differences among patients with 25~29 LNs, 30~34 LNs, and ≥35 LNs examined. And we suspected that the cutoff level was 25 LNs for stage II. For the patients with stage III disease, there were remarkable survival differences among patients with 0~9 LNs, 10~14 LNs, 15~19 LNs, 20~24 LNs, 25~29 LNs, and 30~34 LNs (*P* < 0.05). However, there were no survival differences between patients with 30~34 LNs and ≥35 LNs. So the cutoff level was regarded as 30 LNs. Thus, it was that the cut-point analysis yields the greatest variance of survival rate difference at the levels of 15 LNs (stage I), 25 LNs (stage II), and 30 LNs (stage III). 

## 4. Discussion

It is generally accepted that a higher survival rate benefits from the standardized pattern of lymph node dissection [13–15]. Many studies have shown that the number of dissected LNs is closely related to the postoperative pathologic staging and prognostic assessment. A sufficient number of lymph nodes dissected could improve staging reliability and prognostic assessment accurately [16, 17]. Sun et al. [18] reviewed 2159 patients with gastric cancer who had undergone gastrectomy with curative intent. They found that closed linear correlations were observed between the number of retrieved LNs and the number of metastatic nodes. Meanwhile, on account existence of lymph node micrometastases [19–21], the result of exploration for lymph node metastases by routine H&E staining may not exactly reflect the prognosis. As a result, better long-term survival has been observed with higher total LNs, showing the contribution of sufficient lymphadenectomy toward reducing the positive and micrometastasic lymph nodes. According to our data, the median number of total LNs examined was 23 (mean 23.1 ± 8.6) per patient. There was a significant correlation between the number of metastatic lymph nodes and retrieved nodes according to the Pearson's correlation test (*P* < 0.05). We thus conclude that a proper increment of the dissected LN count contributes to the reduction of the number of residual tumor cells. On the other hand, a research result from Italy has pointed out that the number of LNs dissected emerged as one of the most important prognostic indicators [22]. In our present study, the number of dissected LNs was modeled as a common variable. In the multivariate survival analysis, the number of dissected LNs, as well as depth of invasion and lymph node involvement, was one of the independent predictors of survival. In addition, the number of dissected LNs is the most important predictor of survival that can be influenced by the surgeons, especially for the patients without preoperative chemotherapy and underwent a curative resection (R0). Furthermore, a preoperative differential diagnosis between mucosal and deeper gastric cancer is difficult, and it is hard to localize or predict the extension of lymph node metastasis by the current available techniques. And a complete surgical resection with an en bloc LN dissection is still an important procedure to improve survival for gastric cancer. Therefore, the authors propose that an adequacy number of dissected LNs are not only a demand for accurate staging, but also a surrogate marker related to the quality of gastric cancer surgery; it reflects the extent of LN dissection and significance for improving survival in patients with gastric cancer.

In 2003, Le Voyer et al. [23] published a secondary analysis of intergroup trial INT-0089. They emphasized the importance of the number of LNs examined determining prognosis on colon cancer. Additionally, they believed that staging accuracy and survival were improved with increasing nodal examination and analysis. However, there was no international multicentre prospective randomized study on the impact of the removed nodes on outcome in patients with gastric cancer. Smith et al. [9] reviewed T1-3N0-1 gastric cancer with curative intent. They found that in every stage subgroup, overall survival was highly dependent on the number of LNs examined. The higher the number of LNs examined, the better the resulting postgastrectomy survival would be. Schwarz and Smith [10] believed that the results speak for a therapeutic benefit as a result of extended lymph node dissection, even in patients with more advanced (stage III and IV) yet resectable gastric cancer. The results in our present study also showed that, with comparable T category, the overall survival was significantly different (*P* < 0.05), and always in favor of the higher LN counts. From the investigation of the linear projection model, a statistical significant survival improvement based on increasing LNs counts was obvious for stage II and stage III subgroups analyzed. So, if patients were estimated with more advanced gastric cancer preoperation, more dissected LNs should be achieved during the operation. Dissection of much more lymph nodes with the increased stage may improve long-term survival of gastric cancer patients. 

The NCCN Clinical Practice Guidelines in Oncology-Gastric Cancer Guideline requires surgeons to make a preoperative staging via endoscopic ultrasonography, abdominal ultrasonography, computed tomography (CT), and so on preoperation, in order to choose different therapies for patients with different stages. In this paper, we expected the surgeons to estimate the TNM stage before or during the operation and choose the different scopes of LN dissection to obtain sufficient number of dissected LNs. However, it is yet unclear whether sufficient lymph node dissection is needed for staging or better prognosis. How many nodes should be removed after the radical distal gastrectomy for different stages which lead a better survival? Lee et al. [24] showed that patients with gastric cancer should undergo adequate lymphadenectomy to permit examination of ≥15 LNs, which would allow accurate identification of prognostic variables. Removal of ≥15 LNs is associated with higher survival rate for patients with this disease. Another study from Marubini et al. [25], however, suggested that removal of 15 LNs is still insufficient, an extended lymphadenectomy may always be preferable, and the risk of long-term death tends to decrease when the number of resected lymph nodes increases to about 25. Based on the results of the cut-point analysis, we consider that the number of dissected LNs could help us to evaluate the patients' prognosis, and removal of 15 LNs for stage I, 25 LNs for stage II, and 30 LNs for stage III is recommended for an adequate LN resection during the curative distal gastrectomy for the patients with gastric cancer, who achieve the best long-term survival outcomes. 

##  Author Contributions

C.-M. Huang and J.-X. Lin conceived of the study, analyzed the data, and drafted the paper1; C. H. Zheng helped revise the paper critically for important intellectual content; P. Li, J.-W. Xie, and J. B. Wang helped collect data and design the study. All the authors read and approved the final paper. 

## Figures and Tables

**Table 1:** Clinical characteristics of the 634 patients.

Characteristics	Patients (*n* = 634)
Gender	
Male	467 (73.6)
Female	167 (26.4)
Age (years)	56.1 ± 12.1 (range, 22~87)
Tumor diameter (cm)	4.3 ± 1.7 (range, 0.5~15)
Tumor location	
Lower	521 (80.8)
Middle	71 (11.2)
Lower and middle	42 (8.0)
Digestive tract construction	
Billroth I	469 (74.0)
Billroth II	148 (22.3)
Roux-en-Y	17 (3.7)
Pathology	
Differentiated	140 (22.1)
Undifferentiated	494 (77.9)
Depth of invasion	
pT1	118 (18.6)
pT2	106 (16.7)
pT3	68 (10.7)
pT4a	342 (54.0)
pN category	
pN0	195 (30.8)
pN1	115 (18.1)
pN2	117 (18.5)
pN3	207 (32.6)
Number of resected LNs	
0~9 LNs	32 (5.0)
10~14 LNs	75 (11.8)
15~19 LNs	111 (17.5)
20~24 LNs	161 (25.4)
25~29 LNs	118 (18.6)
30~34 LNs	78 (12.3)
≥35 LNs	59 (9.4)
TNM stage	
I	133 (21.0)
II	165 (26.0)
III	336 (53.0)

**Table 2:** Univariate analysis of variables for patients with curative distal gastrectomy.

Characteristics	*N*	5-year survival (%)	*χ*2	*P*
Gender			<0.001	0.991
Male	467	58.1		
Female	167	56.3		
Age (years)			0.641	0.423
<60	370	58.9		
≥60	264	56.0		
Tumor diameter (cm)			4.409	0.036
≤4	343	61.0		
>4	291	53.6		
Tumor location			5.693	0.058
Lower	521	57.7		
Middle	71	63.7		
Lower and middle	42	45.4		
Digestive tract construction			5.508	0.064
Billroth I	469	60.7		
Billroth II	148	48.5		
Roux-en-Y	17	51.0		
Pathology			9.135	0.003
Differentiated	140	68.1		
Undifferentiated	494	54.6		
Depth of invasion			65.354	0.000
pT1	118	90.6		
pT2	106	67.2		
pT3	68	60.1		
pT4a	342	42.9		
pN category			116.863	0.000
pN0	195	83.3		
pN1	115	63.6		
pN2	117	44.5		
pN3	207	31.2		
Number of resected LNs			65.288	0.000
0~9 LNs	32	30.9		
10~14 LNs	75	37.2		
15~19 LNs	111	51.6		
20~24 LNs	161	59.7		
25~29 LNs	118	62.0		
30~34 LNs	78	69.6		
≥35 LNs	59	77.7		
Adjuvant chemotherapy*			1.829	0.117
Yes	382	51.0		
No	134	47.8		

**Table 3:** Multiple stepwise regression analysis with the Cox proportional hazards model.

Characteristics	*β*	SE	Wald	*P*	RR	95% CI
Tumor diameter	−0.042	0.133	0.121	0.689	0.940	0.754–1.317
Pathology	−0.012	0.154	0.006	0.933	0.987	0.725–1.315
Depth of invasion			22.738	0.000		
pT2 versus pT1	0.684	0.258	8.754	0.009	2.015	1.126–3.570
pT3 versus pT1	0.953	0.250	15.547	0.000	2.943	1.681–4.524
pT4a versus pT1	1.253	0.244	21.636	0.000	3.043	1.981–5.158
pN category t			119.502	0.000		
pN1 versus pN0	0.513	0.156	12.244	0.000	2.022	1.401–2.873
pN2 versus pN0	1.364	0.186	48.598	0.000	4.302	3.423–5.966
pN3 versus pN0	2.196	0.237	89.746	0.000	10.379	6.587–18.846
Number of resected LNs	−0.425	0.041	106.996	0.000	0.625	0.588–0.736

*β*: Coefficient of regression.

**Table 4:** Five-year overall survival by stage subgroups and total number of resected LNs.

Subgroup	*N*	Number of resected LNs [*n*, OS (%)]							*P*
		0~9	10~14	15~19	20~24	25~29	30~34	≥35	
Depth of invasion									
pT1	118	6 (62.5)	15 (80.0)	27 (88.9)	29 (93.1)	18 (88.9)	12 (91.7)	11 (100.0)	0.025
pT2	106	7 (28.6)	11 (27.3)	18 (66.7)	28 (75.0)	26 (73.1)	10 (90.0)	8 (87.5)	0.000
pT3	68	3 (33.3)	5 (40.0)	13 (53.8)	15 (60.0)	9 (66.7)	10 (70.0)	11 (72.7)	0.005
pT4a	342	16 (20.2)	44 (23.9)	53 (31.5)	89 (44.4)	65 (50.7)	46 (55.1)	29 (69.0)	0.000

NA: Not applicable; OS: Overall 5-year survival rate.

**Table 5:** Projected numeric total LNs impact on 5-year overall survival.

Stage subgroup	Patients (*n*)	*β*	Baseline projected 5-year survival (0 LN examined), %	For every 10 extra LNs examined, survival improved by (%)	*P *value
I	133	0.846	43.5	13.9	0.061
II	165	0.923	13.6	18.7	0.025
III	336	0.964	2.0	15.5	0.003

Total	634	0.952	20.4	13.1	0.001

*β*: Coefficient of regression.

**Table 6:** Pairwise comparisons of overall survival of patients with different number of dissected LNs after surgery by Kaplan-Meier method.

Stage	Removed LNs	*P* value						
		0~9	10~14	15~19	20~24	25~29	30~34	≥35
I	0~9	—	0.004	0.000	0.000	0.000	0.000	0.000
	10~14	0.004	—	0.046	0.003	0.035	0.040	0.012
	15~19	0.000	0.046	—	0.121	0.375	0.268	0.116
	20~24	0.000	0.003	0.121	—	0.702	0.631	0.540
	25~29	0.000	0.035	0.375	0.702	—	0.387	0.351
	30~34	0.000	0.040	0.268	0.631	0.387	—	1.0
	≥35	0.000	0.012	0.116	0.540	0.351	1.0	—

II	0~9	—	0.889	0.132	0.001	0.000	0.000	0.000
	10~14	0.889	—	0.124	0.000	0.000	0.000	0.000
	15~19	0.132	0.124	—	0.009	0.000	0.000	0.000
	20~24	0.001	0.000	0.009	—	0.044	0.145	0.028
	25~29	0.000	0.000	0.000	0.044	—	0.899	0.963
	30~34	0.000	0.000	0.000	0.145	0.899	—	0.751
	≥35	0.000	0.000	0.000	0.028	0.963	0.751	—

III	0~9	—	0.131	0.107	0.010	0.000	0.000	0.000
	10~14	0.131	—	0.758	0.212	0.033	0.012	0.000
	15~19	0.107	0.758	—	0.087	0.011	0.000	0.000
	20~24	0.010	0.212	0.087	—	0.136	0.026	0.001
	25~29	0.000	0.033	0.011	0.136	—	0.042	0.030
	30~34	0.000	0.012	0.000	0.026	0.042	—	0.198
	≥35	0.000	0.000	0.000	0.001	0.030	0.198	—
